# Bacteria assisted green synthesis of copper oxide nanoparticles and their potential applications as antimicrobial agents and plant growth stimulants

**DOI:** 10.3389/fchem.2023.1154128

**Published:** 2023-04-07

**Authors:** Deepak Singh, Devendra Jain, Deepak Rajpurohit, Gajanand Jat, Himmat Singh Kushwaha, Abhijeet Singh, Santosh Ranjan Mohanty, Mohammad Khalid Al-Sadoon, Wajid Zaman, Sudhir K. Upadhyay

**Affiliations:** ^1^ Department of Molecular Biology and Biotechnology, Maharana Pratap University of Agriculture and Technology, Udaipur, India; ^2^ Department of Soil Science and Agricultural Chemistry, Maharana Pratap University of Agriculture and Technology, Udaipur, India; ^3^ Material Research Centre, Malviya National Institute of Technology, Jaipur, India; ^4^ Department of Biosciences, Manipal University Jaipur, Jaipur, India; ^5^ All India Network Project on Soil Biodiversity-Biofertilizers, ICAR-Indian Institute of Soil Science, Bhopal, India; ^6^ Department of Zoology, College of Science, King Saud University, Riyadh, Saudi Arabia; ^7^ Department of Life Sciences, Yeungnam University, Gyeongsan, Republic of Korea; ^8^ Department of Environmental Science, V. B. S. Purvanchal University, Jaunpur, India

**Keywords:** novel bacterial isolate, 16s-rDNA sequencing, CuO-NPs-green synthesis, confirmatory tests, antimicrobial and plant growth-promoting activity

## Abstract

Copper oxide nanoparticles (CuO-NPs) have piqued the interest of agricultural researchers due to their potential application as fungicides, insecticides, and fertilizers. The *Serratia* sp. ZTB29 strain, which has the NCBI accession number MK773873, was a novel isolate used in this investigation that produced CuO-NPs. This strain can survive concentrations of copper as high as 22.5 mM and can also remove copper by synthesizing pure CuO-NPs. UV-VIS spectroscopy, DLS, Zeta potential, FTIR, TEM, and XRD techniques were used to investigate the pure form of CuO-NPs. The synthesized CuO-NPs were crystalline in nature (average size of 22 nm) with a monoclinic phase according to the XRD pattern. CuO-NPs were found to be polydisperse, spherical, and agglomeration-free. According to TEM and DLS inspection, they ranged in size from 20 to 40 nm, with a typical particle size of 28 nm. CuO-NPs were extremely stable, as demonstrated by their zeta potential of −15.4 mV. The ester (C=O), carboxyl (C=O), amine (NH), thiol (S-H), hydroxyl (OH), alkyne (C-H), and aromatic amine (C-N) groups from bacterial secretion were primarily responsible for reduction and stabilization of CuO-NPs revealed in an FTIR analysis. CuO-NPs at concentrations of 50 μg mL^−1^ and 200 μg mL^−1^ displayed antibacterial and antifungal activity against the plant pathogenic bacteria *Xanthomonas* sp. and pathogenic fungus *Alternaria* sp., respectively. The results of this investigation support the claims that CuO-NPs can be used as an efficient antimicrobial agent and nano-fertilizer, since, compared to the control and higher concentrations of CuO-NPs (100 mg L^−1^) considerably improved the growth characteristics of maize plants.

## 1 Introduction

Nanotechnology research is the most active research region in contemporary materials science (Singh et al., 2018; Rajput et al., 2021a; Bhavyasree and Xavier, 2022). Nanomaterials synthesis through conventional physical and chemical methods has several adverse features *viz.*, critically high pressure and temperature conditions, utilization of expensive and hazardous chemicals, a longer reaction time and absorbance of toxic by-products on nanomaterial surface (Buazar et al., 2019; Sukumar et al., 2020). Properties of NPs determined by their size, shape, composition, crystalline, and structure (Sharma et al., 2020; Hidangmayum et al., 2022; Rajput et al., 2022). Recent years have seen a significant increase in the significance of green synthesis techniques for nanomaterials, making it one of the very popular methods in modern material sciences (Sukhwal et al., 2017; Mahboub et al., 2022).

Green synthesis has become one of the most preferred methods to overcome the adverse effects physical and chemical synthesis such as critical conditions of temperature and pressure, expensive and toxic chemicals, long reflux time of reaction, toxic by-products *etc.* (Sukhwal et al., 2017; Jain et al., 2020). Metal-tolerant bacteria are important nano-factories that not only accumulates and also detoxify heavy metals due to the various mechanism, *i.e.*, reductase enzymes, EPS, *etc.*, to reduce metal salts to nanomaterials (Jain et al., 2012; Jain et al., 2020; Garg et al., 2022). The nanomaterial synthesis using plant extracts may be easier than microbial synthesis however the microbial synthesis is more cost-effective and freer from any seasonal and plant growth stage variation.

Inorganic metal oxide NPs, *viz.*, CuO, ZnO, MgO, TiO_2_, SiO_2_, *etc.*, with significant antimicrobial features as well as their selective toxicity, point to potential applications of these materials in medical devices and diagnostics, therapeutics, and nanomedicine against human pathogens (Mohsen and Zahra, 2008; Sobha et al., 2010; Jain D. et al., 2022). These inorganic oxide NPs are beneficial as antibacterial agents because they are more effective against resistant pathogens. According to Makhluf et al. (2005), crystalline structure and particle shape of nanomaterials have relatively little effect on antibacterial behavior, but a high concentration of smaller-size nanoparticles with a higher surface area does.

The simplest copper compound in the family is copper oxide, which has a variety of possibly practical physical characteristics (Buazar et al., 2019). Copper oxide (CuO) has drawn more interest than other nanomaterials because of its distinctive qualities, which include stability, conductivity, catalytic activity, and anticancer and antibacterial activities. Copper oxide nanoparticles (CuO-NPs) are receiving more attention owing to their availability and lower cost when compared to more costly and noble metals like gold and silver, as well as their effective potential for application as microbial agents (Sankar et al., 2014). Among them, CuO-NPs has drawn a lot of attention in research areas including solar cells, biodiesel, photocatalysis, water pollutant removal, supercapacitors, and electrocatalysis owing to their desired qualities, such as cheap cost, non-toxicity, and ease of manufacturing (Grigore et al., 2016).

By preventing the growth of bacteria, fungi, viruses, and algae, CuO-NPs have important antimicrobial qualities (Amin et al., 2021; Bukhari et al., 2021). Furthermore, compared to other organic antimicrobials like silver and gold, nanoscale copper oxide has a longer shelf life. According to Keabadile et al. (2020) green synthesis of CuO-NPs with acceptable physio-chemical characteristics has previously been performed with several microbial precursors as reductants. However, very little study has been done on the synthesis of CuO-NPs employing bacteria that are copper-resistant. Hence, the current investigation was conducted to tackle this issue and build a bacteria-assisted synthesis of CuO-NPs and assessment of their antimicrobial and plant growth stimulating activities.

## 2 Materials and methods

### 2.1 Source, minimum inhibitory concentration, and molecular identification of copper-tolerant bacteria

The maximum copper tolerance concentration (MTC) was determined on LB agar medium (in triplicate) having an increased concentration of CuSO_4_ (2.5–25 mM), and the MTCs were noted from the concentration of CuSO_4_ at which the isolate failed to demonstrate growth. The different bacterial isolates were utilized in this study taken from our lab, which were isolated from Zn-Pb ore mine tailings areas of Zawar mines in Udaipur, Rajasthan, India (Jain et al., 2020). According to a previously illustrated method, the 16S rDNA region was amplified and sequenced to perform molecular characterization of copper-tolerant bacteria (Janda and Abbott, 2007).

### 2.2 Bacterial-assisted synthesis of copper oxide nanoparticles

The synthesis of CuO-NPs was borne out by using copper (Cu) tolerant bacterial isolate (ZTB29) with little modification technique of earlier published (John et al., 2021). The bacterial strain that showed the highest tolerance against copper ion, was inoculated in LB medium (100 mL) and incubated at 28°C with 150 rpm. After 24 h, 5 mM CuSO_4_.5H_2_O was dropped into the bacterial culture and incubated for 48 h at 28°C until the solution color changed from blue to green. This combination was then centrifuged at 4,000 rpm for 20 min at 4°C to separate the bacterial cell pellet, and the CuO-NPs were produced by centrifuging the residual supernatant at 14,000 rpm for 15 min at 4°C. The obtained CuO-NP pellet was washed twice with deionized water, dried at 80°C in an oven and used for further characterization. A control experiment without copper-tolerant bacteria was also done and upon inclusion of 5 mM CuSO_4_.5H_2_O, the color change was not seen which states no nanoparticles formation.

### 2.3 Characterization of CuO-NPs

CuO-NPs were primarily characterized using UV-Vis absorption scanning at 200–1,000 nm using a nanophotometer (Make: Implen, Germany) as the method outlined by Davaeifar et al. (2019). Dynamic Light Scattering (DLS) and Zeta potential were performed by the earlier described method (Rajput et al., 2021b) by using Malvern zeta-sizer nanoseries (United Kingdom). The FTIR spectroscopy (Perkin Elmer) was performed for Cuo-NPs (in KBr pellets) in the 4,100–400 cm^−1^ range (Garg et al., 2022). Around 10 µL of CuO-NPs dispersed in milli Q water were placed onto carbon-coated copper TEM grid for transmission electron microscopy (Tecnai G220 (FEI) S-Twin 200kv) (Sukhwal et al., 2017). The dried powder of CuO-NPs was further characterized by XRD (X’Pert Pro X-ray diffractometer, PAN analytical BV) with Cu Kα radiation set with 40 kV and 30 mA (Sukhwal et al., 2017).

### 2.4 Antimicrobial activities of CuO nanoparticles

Antibacterial activities of bacterial-assisted CuO-NPs were studied by both disc diffusion method and well diffusion using LB agar medium against plant pathogenic bacteria *Xanthomonas* sp. Briefly, 1 mL bacterial suspension (>10^7^ CFU mL^−1^) was spread by spreader on LB agar Petri-plates, and in disc diffusion method, the sterile filter paper disk, dipped in a known concentration of CuO-NPs was placed on LB agar plates whereas, in well diffusion method, 5 mm wells (prepared by sterile cork-borer on LB agar Petri-plates) were loaded with CuO-NPs and incubated for inhibition zone development (Jain et al., 2020). The antifungal activities of CuO-NPs were investigated by using the poisoned food technique and spore germination test. The radial mycelia growth of test fungi *Alternaria* sp. was recorded on PDA containing different concentrations of CuO-NPs (50, 75, 100, 150, and 200 μg mL^−1^). PDA plates without CuO-NPs were used as a control. These plates were kept for incubation at 25°C until full radical growth was observed in the control. The different concentration of CuO-NPs was used as per the CRD design in triplicate and the significant difference among treatment were determined by Turkey–Kramer HSD test at *p* = 0.05.

### 2.5 *In vitro* studies of CuO-NPs on the growth of maize

The experimental pot was filled with agricultural soil supplemented with sterile planting mixture, seeded with maize seed (PRATAP-3), and placed inside the plant growth chamber (humidity: 60%, light intensity: 750 μmol/m^2^s with 15 h light and 9 h dark conditions at 25°C–20°C). Seven days old maize seedlings were treated with CuO-NPs concentrations *viz.*0, 25, 50, 75, 100, 200, and 300 mg L^−1^ (in Hoagland solution) as foliar spry. The shoot length (cm), root length (cm), chlorophyll content (SPAD-502 + Chlorophyll Meter, Spectrum Technologies, India), Copper content [atomic absorption spectroscopy (AAS), Make: Electronics co. India Ltd. Modal no. AAS4141] was studied in 21 days old seedlings (Garg et al., 2022).

## 3 Result

### 3.1 Source, screening of MTC against copper and molecular identification of potent copper-tolerant bacteria

**FIGURE 1:**
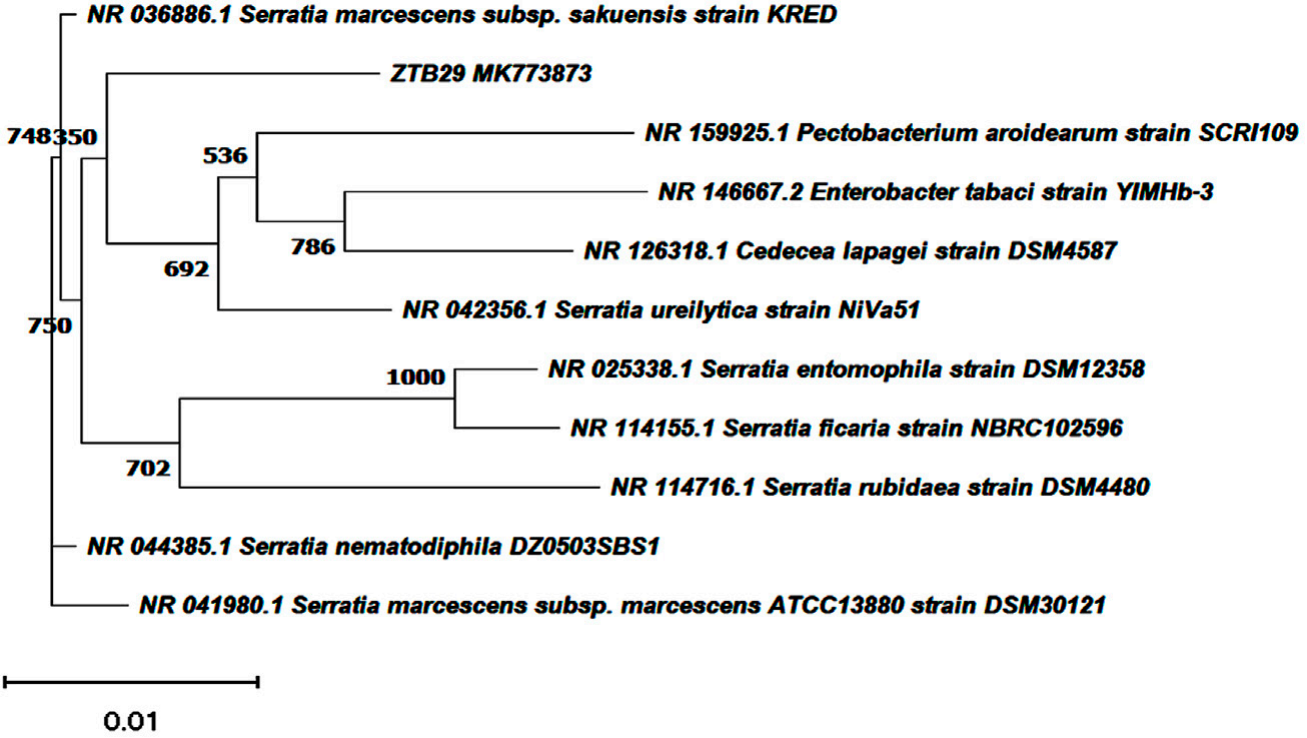
Neighbourhood joining tree showing the polygenetic relationship of copper tolerant bacterial strain ZTB29 *Serratia* sp. (NCBI Accession: MK773873).

The bacterial isolates ZTB15, ZTB24, ZTB28, and ZTB29 were tested for their maximum copper (CuSO_4_) tolerance levels in nutritional broth and observed Minimum inhibitory concentration (MIC). The bacterial isolate ZTB29 had a very maximum MIC of 22.5 mM copper in the medium and was able to withstand high doses of copper in the current experiment (Supplementary Table S1). A further selection of the ZTB29 strain was made for the bacterially aided synthesis of copper oxide CuO-NPs. The ZTB-29 isolate’s 16S rRNA gene was sequenced in its entirety and put into nucleotide-nucleotide BLAST analysis. The strains’ similarity and matches to previously published bacterial rDNA sequences allowed scientists to identify them as *Serratia* sp. (Figure 1). The ZTB29 nucleotide sequence was deposited to NCBI with the accession MK773873. The detailed biochemical, plant growth promoting and other physiological attributes of the ZTB29 strain were summarized in (Supplementary Table S2) which enables the ZTB29 strain to not only bioremediate excess copper but also to promote plant growth.

### 3.2 ZTB29 assisted copper oxide nanoparticles synthesis and its confirmatory examination

**FIGURE 2:**
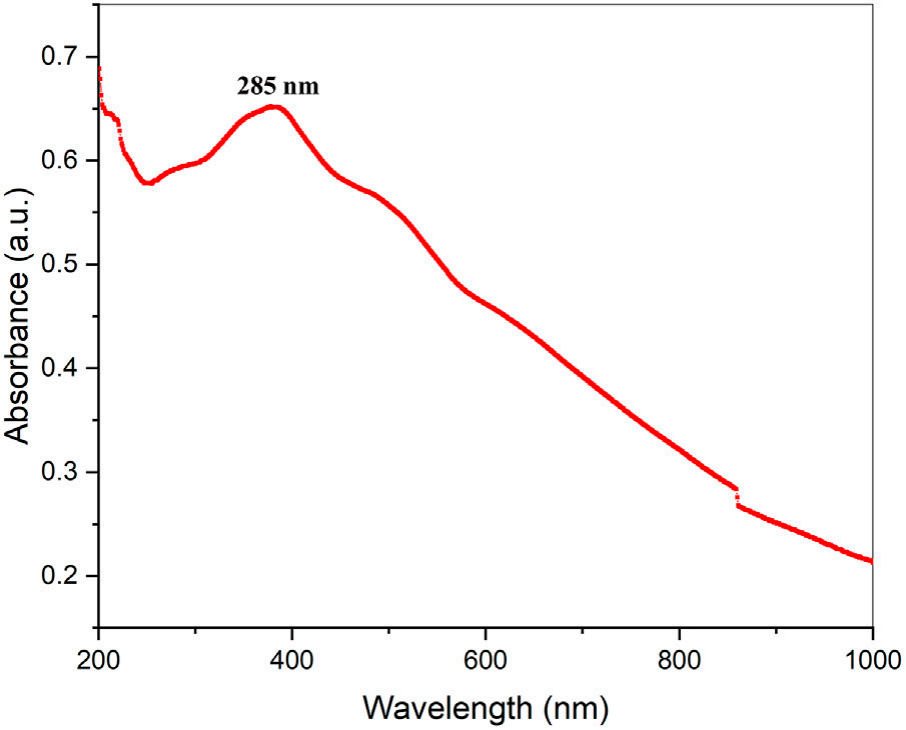
UV-Vis absorption spectrum of biogenic CuO nanoparticles.

The easily observed synthetic bacterial growth in the bottom of the flask demonstrated the reaction between the bacterium and copper sulfate, the precursor salt. The starting solution’s color changed from light blue to green when 5 mM copper sulfate was added drop by drop to the bacterial suspension, indicating the production of CuO-NPs. The greatest absorbance of 285 nm by using UV-visible spectroscopy was observed, indicating that copper sulfate (which does not produce any absorbance at 285 nm: Supplementary Figure S1), the starting material, was converted to CuO-NPs, as shown in (Figure 2).

**FIGURE 3:**
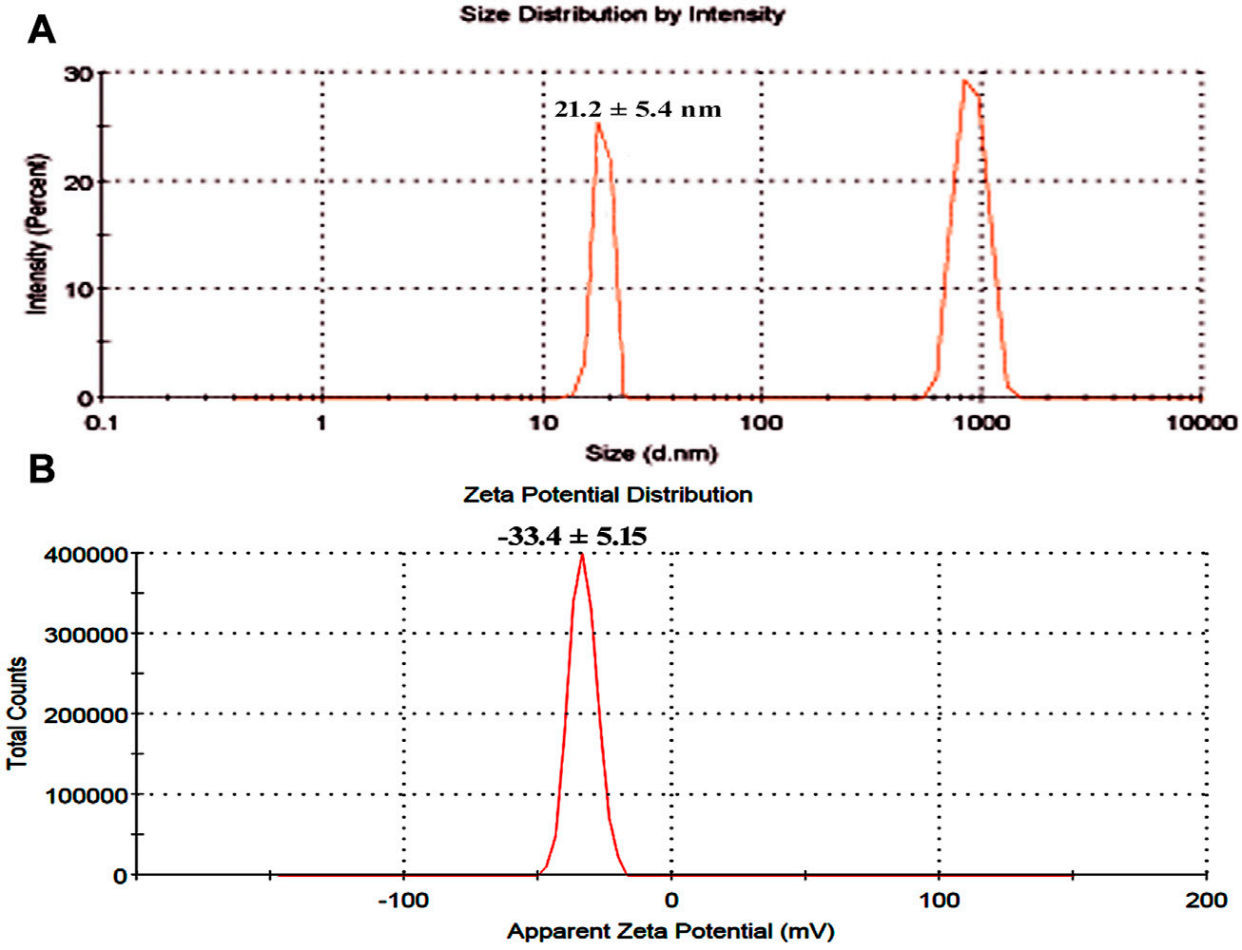
**(A)** Particle size determination using dynamic light scattering **(B)** Zeta potential analysis of bacterial assisted CuO nanoparticles.

The surface charge, size distribution, and potential stability of the nanoparticles contained in a liquid were characterized using dynamic light scattering (DLS) and zeta potential, respectively. Particles in the solution ranged in size from 15 nm to 30 nm and were homogeneous in size. The average CuO-NPs particle size was 21 ± 5.4 nm which was created with a homogenous dispersion (Figure 3A). The TEM investigations provided strong support for the DLS findings. The presence of bacterial cell artifacts or the agglomeration of nanoparticles may be responsible for the second large-size distribution peak at about 1,000 nm. The zeta potential’s magnitude (−30 mV to +30 mV) determines the stability and primarily depends upon the surface charge of the generated nanomaterials. The produced nanoparticles have a Zeta potential of −15.4 mV, which demonstrates that they were quite stable at ambient temperature (Figure 3B). The similar zeta potential value was observed even after 1 year of synthesis with CuO-NPs suggesting CuO-NPs were stable for 1 year or more. Zeta potential with a negative value indicates a strong repelling force between the particles, which inhibits agglomeration.

**FIGURE 4:**
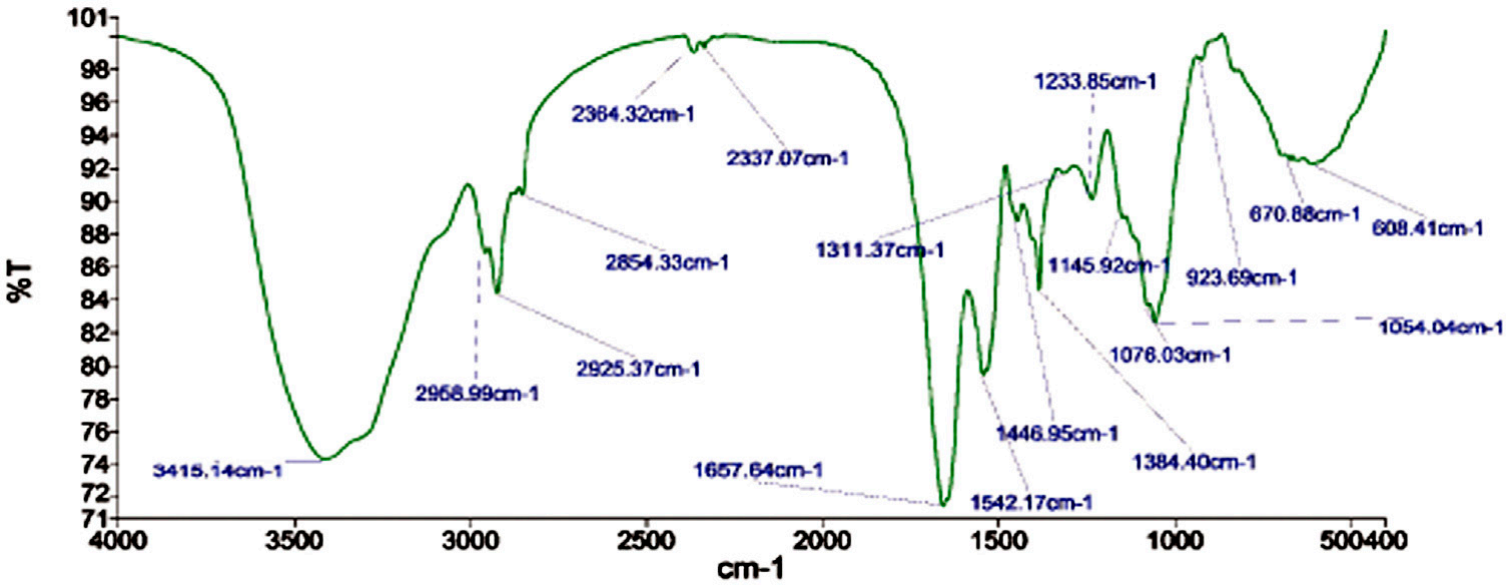
FTIR analysis of biogenic CuO nanoparticles.

**FIGURE 5:**
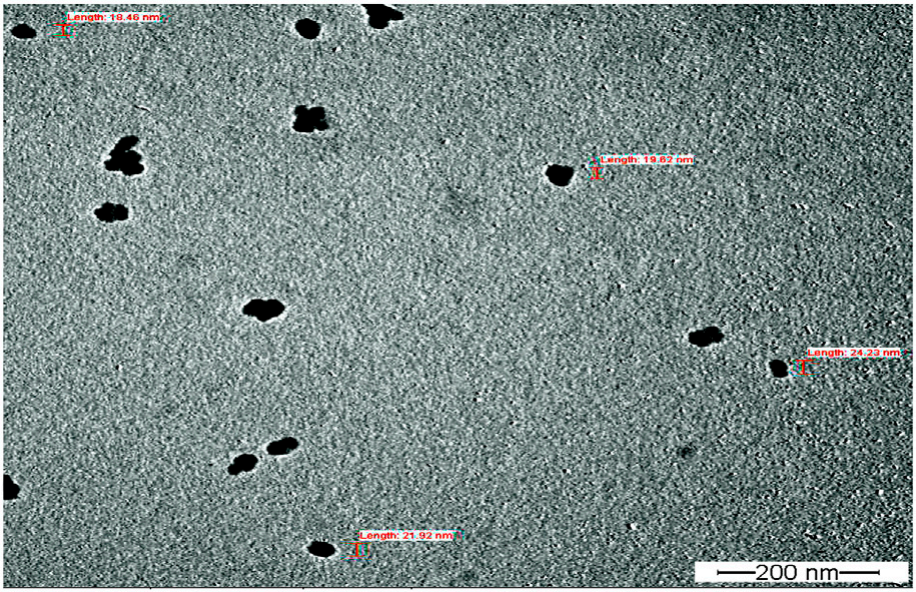
TEM analysis of biogenic CuO nanoparticles.

Fourier transform infrared spectroscopy (FTIR) technique was utilized to recognize the occurrence of different functional groups found in a sample. Depending on the infrared absorption range 600–4,000 cm^−1^ in FTIR analysis, the absorbance range 3,200–3,550 cm^−1^ is indicated for O-H stretching, 2,371 cm^−1^ observance for O=C=O stretching, 1,624 cm^−1^ observance for C=C stretching, 1,058 cm^−1^ observance for C-OH stretching, 1,377 cm^−1^ observance for the existence of CO_2_ when compared with the standard database. The 608 cm^−1^ vibration attributed to CuO formation confirms the synthesis of pure CuO nanostructures. FTIR study revealed that the carboxyl (C=O), hydroxyl (OH), amine (NH), alkyne (C-H), thiol (S-H), ester (C=O) and aromatic amine (C-N) groups from the bacterial secretion are responsible for the copper reduction and CuO-NPs stabilization (Figure 4). The details of the different FTIR peaks observed in bacteria assisted CuO-NPs and the bacterial extract used for CuO-NPs synthesis were described in the Supplementary Table S3 and Supplementary Figure S2. The CuO-NP’s size and shape were studied using TEM. TEM analysis revealed the formation of different shapes of copper oxide nanostructures (Figure 5). It was evident from TEM studies that CuO-NPs were polydisperse and spherical which were free from agglomeration. The particles were in the size range of 20–40 nm with 28 nm average particles size.

**FIGURE 6:**
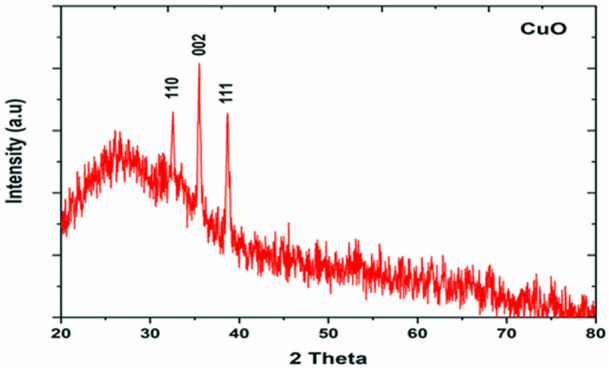
XRD analysis of bacterial assisted CuO nanoparticles.

X-ray diffraction (XRD) was performed to study the phase (structure) and purity (composition) of the biosynthesized CuO-NPs using copper-tolerant bacteria. The XRD pattern (Figure 6) depicted the creation of pure and crystalline CuO-NPs. The peaks at 2 = 32.548, 35.466, and 38.769 were assigned to the (110) (002) and (111) reflection lines of monoclinic CuO-NPs compared to JCPDS file No. 01-080-1268. The average crystallite size calculated based on the Scherrer technique for synthesized CuO-NP was 22 nm.

### 3.3 Antimicrobial activities of CuO-NPs

**FIGURE 7:**
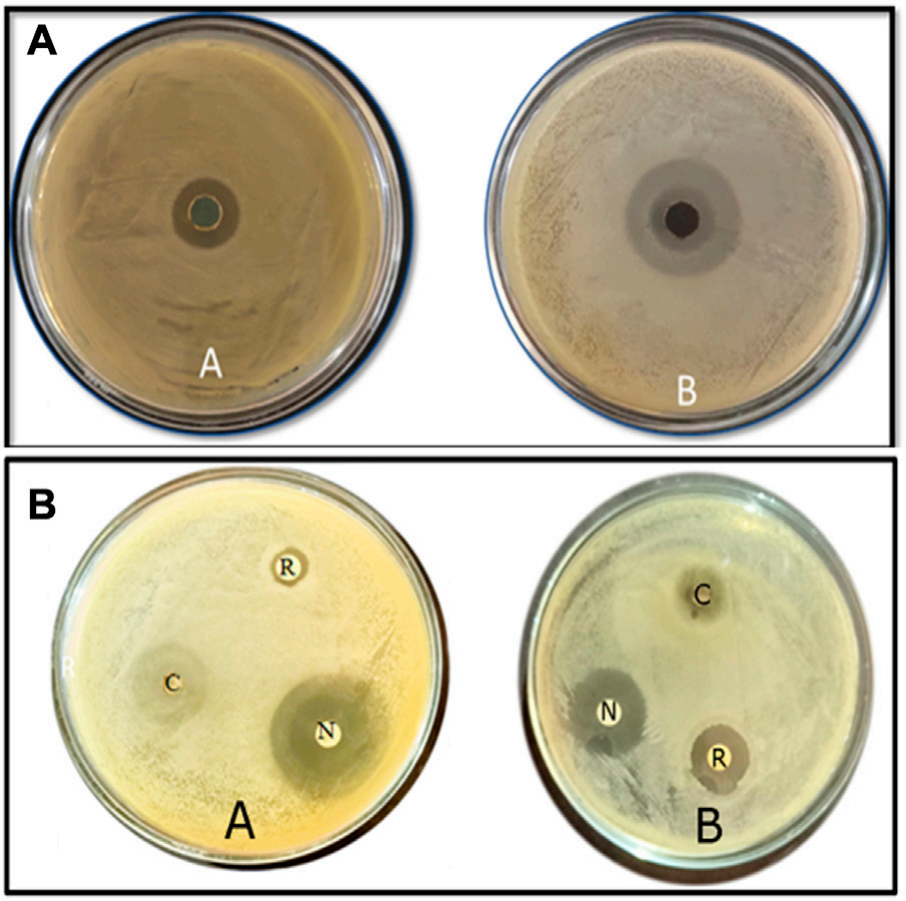
Antibacterial activities of biogenic CuO nanoparticles at 50 μg mL^−1^ the concentrationagainst **(A)** *Xanthomonas* Sp. **(B)** *E. coli* using (a) well diffusion method (b) disc diffusion method along with Rifampicin 5 μg mL^−1^ and Neomycin 30 μg mL^−1^.

**FIGURE 8:**
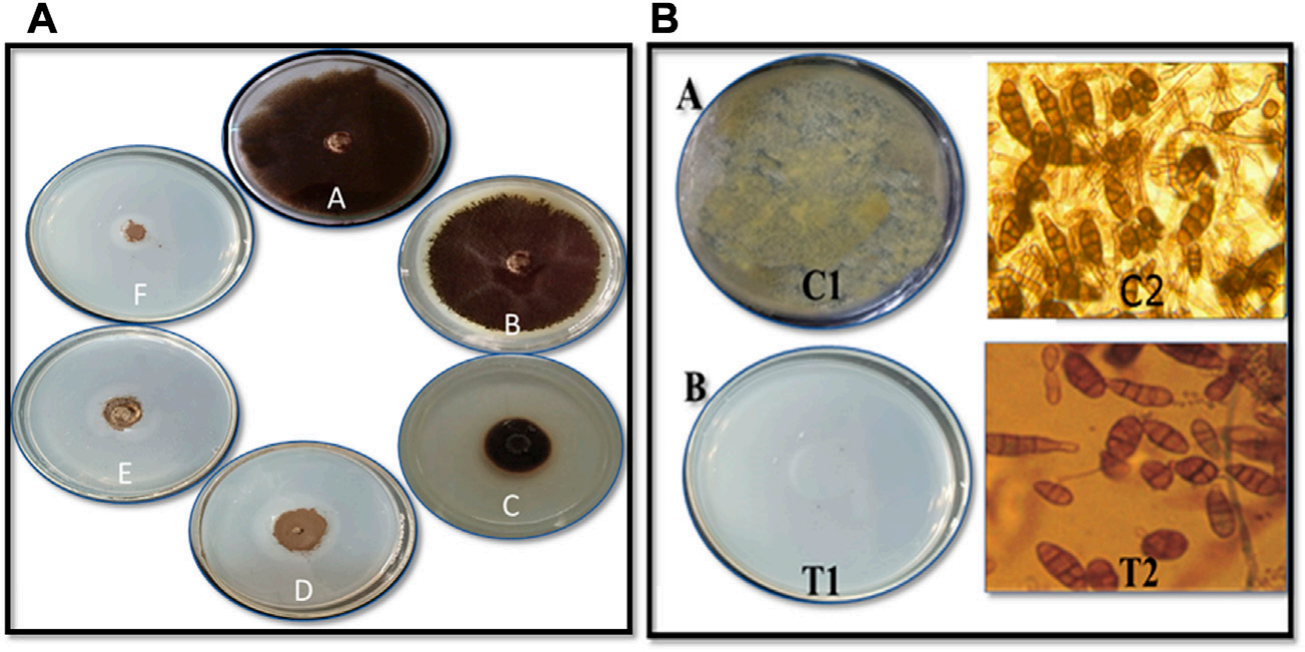
Antifungal activity of CuO nanoparticles against *Alternaria*sp. on **(A)** mycelial growth inhibition by poisoned food technique (A) Control (B) 50 μg mL^−1^ (C) 75 μg mL^−1^ (D) 100 μg mL^−1^ (E) 150 μg mL^−1^ and (F) 200 μg mL^−1^CuO nanoparticles **(B)** Spore germination inhibition by **(A)**Pour plate technique C1 - Crude spore suspension and T1 - 200 μg mL^−1^CuO nanoparticles **(B)** Microscopic studies C2: Control, T2: 200 μg mL^−1^CuO nanoparticles.

The CuO-NPs (50 μg mL^−1^) showed significant antibacterial activity by generating an inhibition zone in well diffusion assay (Figure 7A). The disc contacting 50 μg mL^−1^ CuO-NPs demonstrated antibacterial activity against *Xanthomonas* sp. as it showed a clear inhibition zone (Figure 7B), which was higher compare to Neomycin (30 μg mL^−1^) and lower compare to Rifampicin (5 μg mL^−1^). The highest inhibition of 91% in fungal mycelia and 88% spore germination was detected at the 200 μg mL^−1^ CuO-NPs concentration (Table 1). The rate of mycelia inhibition and spore germination was proportional to CuO-NPs concentration (Figure 8). The results observed in the present study revealed CuO-NP scan be used as an efficient nano fungicide against soil-born fungus.

**TABLE 1:** Effect of varying concentrations of CuO nanoparticles on *in vitro* mycelial growth and spore germination of phytopathogenic fungi *Alternaria* sp.

Treatment (CuO nanoparticles)	Percent inhibition mycelia growth	Percent inhibition spore germination
Control	0.0 ± 0.0_A_	5.33 ± 1.52_A_
50 μg mL^−1^	15.0 ± 1.53_B_	15.0 ± 2.0_B_
75 μg mL^−1^	70.0 ± 1.53_C_	55.0 ± 2.0_C_
100 μg mL^−1^	77.0 ± 5.7_CD_	64.3 ± 2.51_D_
150 μg mL^−1^	81.0 ± 2.0_D_	74.0 ± 1.0_E_
200 μg mL^−1^	91.0 ± 1.52_E_	88.0 ± 2.0_F_

^a^ Each value is mean of 3 replicates from 2 experiments. Mean ± SE, followed by same letter in column of each treatment is not significant difference at *p* = 0.05 by Tukey–Kramer HSD, test, % inhibition rate was calculated compared to the germination of the control (0%).

### 3.4 Influence of CuO-NPs on maize seedling

The shoot and root length, plant biomass, total chlorophyll and copper content were considerably high in the maize plantlet (21 days old) compared to the untreated control plantlet (Table 2). The maximum shoot and root length, biomass and chlorophyll content were observed in 100 mg L^−1^ CuO-NPs application and contributed to plant growth significantly as efficient nano-fertilizers. The CuO-NPs (<100 mg L^−1^) caused significant toxicity to maize seedlings and resulted in decreased growth parameters.

**TABLE 2:** *In vitro* studies on the effect of CuO-NPs on growth of maize seedling (Data are means of three replicates ±SD. Data are recorded after 21 days of germination).

Treatment	Average shoot length (cm)	Shoot dry weight g)	Average root length (cm)	Chlorophyll index (SPAD)	Copper content in maize seedling (ppm)
Control	18.4 ± 1.7	4.4 ± 1.1	12.8 ± 2.1	10.03 ± 2.11	0.041 ± 0.02
T 1	24.5 ± 2.4	7.8 ± 2.3	14.8 ± 3.2	12.33 ± 2.65	0.055 ± 0.08
T 2	28.8 ± 2.8	8.3 ± 1.6	21.6 ± 2.4	13.63 ± 2.49	0.088 ± 0.06
T 3	32.4 ± 2.2	8.4 ± 2.1	24.1 ± 1.9	14.14 ± 2.43	0.092 ± 0.08
T 4	34.2 ± 3.1	10.6 ± 1.8	24.8 ± 2.3	16.86 ± 4.5	0.098 ± 0.09
T 5	29.4 ± 2.3	9.1 ± 1.5	22.7 ± 2.1	13.93 ± 2.8	0.012 ± 0.05
T 6	21.1 ± 1.9	7.5 ± 1.2	15.1 ± 1.7	11.26 ± 2.3	0.015 ± 0.05

## 4 Discussions

The new study could pave the way for bioprospecting for metal-tolerant microorganisms for the quick and easy synthesis of nanoparticles with a variety of applications (Jain et al., 2012; Jain et al., 2020). John et al. (2021) investigated bacterial strain copper tolerance at various CuSO_4_ concentrations, and the bacterial strain *Marinomonas*, which tolerated 5 mM CuSO_4_, was employed to produce copper and CuO-NPs. Similar findings were found in the current investigation. Tiwari et al. (2016) synthesized CuO-NPs from a copper-resistant *Bacillus cereus* isolate that tolerated >10 mM of copper. The *B. cereus* isolate was able to and was identified as *B. cereus* using 16S rDNA amplification and sequencing. The change of color depends on the surface plasmon vibration of the nanoparticles (Abdulhameed et al., 2019). Shantkriti and Rani (2014) observed that the color of the reaction changed from blue to dark green when CuSO_4_ was added to the *Pseudomonas fluorescens* solution, which corroborated the findings. The bacteria-assisted green synthesis of metal and metal oxide nanoparticles is dependent on the bacteria’s ability to remediate harmful metal concentrations by reducing metal ions to nanoparticles (Jain et al., 2020). As a result, copper-tolerant bacteria produce copper and copper oxide nanomaterials by mimicking the natural biomineralization processes that these microbes have adapted to under dangerous copper concentrations (John et al., 2021).

UV-visible absorption spectroscopy can be used to characterize metallic nanoparticles based on surface plasmon resonance (SPR) (Upadhyay et al., 2023). UV-visible spectroscopy (wavelength scan between 200 and 1,000 nm) was used to observe the solution resulting from the bluish-to-greenish color alterations of copper-tolerant bacteria (Zhao et al., 2022). The spectra of CuO-NPs generated employing copper-tolerant bacteria showed pronounced absorption at 285 nm wavelength, confirming the conversion of the starting material (copper sulfate) to the final product (CuO nanoparticles) as shown in Figure 3. Tshireletso et al. (2021) revealed that the UV-VIS absorption spectra of green-produced CuO-NPs from citrus peel extracts resulted in a notable absorbance at 290 nm. Due to surface plasmon resonance, Sankar et al. (2014) found that the UV-Vis spectra of papaya leaf extract medicated CuO-NPs spanned between 250–300 nm. In contrast, the different experiments revealed distinct absorption peaks and spectrums, which could be attributable to different forms of copper and copper oxide nanomaterials and the technology employed for nanomaterial fabrication.

DLS confirmed that the produced CuO-NPs had a homogeneous particle size distribution (15 nm–30 nm) and an average particle size of 21 ± 5.4 nm (Figure 4A), which TEM investigations also validated. The CuO-NPs’ −15.4 mV zeta value clearly demonstrated their fairly stable character, as illustrated in Figure 4B. Nardella et al. (2022) conducted DLS investigations of biosynthesized CuO-NPs and reported a 24.4 nm Z-average diameter, while the zeta potential value, which frequently analyses the stability of nanoparticles, was found to be −16.1 mV, confirming the nanoparticles’ stability. Nagaraj et al. (2019) reported the *Pterolobium hexapetalum* leaf extract-mediated synthesized CuO-NPs and the synthesized nanoparticles were extensively distributed and highly dispersed in the 10–76 nm size range, however, the associated zeta potential was −27.6 mV attributed to moderate stability of nanoparticles.

FTIR study indicated the presence of different compounds from the bacterial secretion involved in the reduction and stabilization of CuO-NPs. Amin et al. (2021) observed the FTIR peaks at 518.4 and 600.1 cm^−1^ (formation of CuO nanostructure and Cu–O stretching), 1,021.14 and 800.58 cm^−1^ (assigned to C–O and C–H bending) and 1,412.3 and 1,636.4 cm^−1^(O–H bending and C=C stretching). John et al. (2021) studied FTIR spectroscopy of CuO-NPs synthesized from marine bacteria indicating the presence of -C=O, –OH, –NH, –CH_2_scissor vibrations of aliphatic compounds and C=C bonds inside the biomolecules suggesting the interaction of these biomolecules with CuO-NPs also observed in the present study.

TEM analysis revealed the formation of polydisperse and roughly spherical CuO-NPS which were free from agglomeration with the 20–40 nm size range (average particle size is 28 nm). The CuO-NPs as water suspension are slightly agglomerated due to their interaction with water and due to such inter-particle interactions viz. van der Waals, electrostatic and magnetic forces, *etc.* Previously, similar results of biogenic CuO-NPs were reported by several studies (Ida et al., 2010; Cheng and Walker, 2010; Chandra et al., 2014; Sagadevan and Koteeswari, 2015; Kimber et al., 2020; Singh et al., 2019) as observed in TEM. John et al*.* (2021) reported the TEM micrographs of CuO-NPs from marine bacteria and reported the synthesis of monodispersed, spherical/ovoidal NPs of 10 nm–70 nm size with ∼40 nm average size. The irregular shape can be attributed to bacterial metabolites on the surface of nanoparticles as stabilizing and reducing agents. Bukhari et al. (2021) reported the *Streptomyces* sp. mediated Cu-NPs synthesis of uniform and spherical nanoparticles (1.72–13.49 nm) in the TEM images. Krishna et al. (2020) synthesized CuO nanoparticles from *Cinnamomum malabatrum* aqueous leaf extract and the TEM revealed spherically shaped CuO-NPs with 11 nm–24 nm size range which was also in close agreement with the present study.

The XRD pattern revealed the pure CuO-NPs were crystalline in nature. Ali et al. (2021) performed XRD of CuO-NPs and the detected peaks in their study confirmed the monoclinic phase of CuO compared to JCPDS card 000021040 which was also seen in the present study. Further, the characteristic crystallite size measured using the Scherrer equation was found to be 24.7 nm also supports the finding of the present study. Buazaret al. (2019) reported that clear and sharp peaks in XRD can be ascribed to the highly crystalline structure of nanomaterials. Similar results of the crystallite size of CuO-NPs in the range of 9–23 nm were solely dependent on the precursor conditions (Tavakoli et al., 2019).

The CuO-NPs exhibited superior antimicrobial activities and have a significant potential to control phytopathogens. Krishna et al. (2020) reported significant antibacterial activities of CuO-NPs against human pathogenic bacteria *viz.*, *Escherichia coli*, *Staphylococcus aureus*, *Pseudomonas aeruginosa* and *Proteus mirabilis* using well diffusion method and similar results were also observed against plant pathogenic bacteria in the present study. Abboud et al. (2014) reported significant antimicrobial activities of CuO-NPs synthesized from the alga extract against *Enterobacter aerogenes* and *S. aureus* and the observed radial diameter of the inhibition zone was 14 and 16 mm, respectively. Bhavyasree and Xavier (2022) extensively reviewed the copper and CuO nanomaterial and their antimicrobial properties and demonstrated the mechanism of antibacterial action which includes mechanical damage, gene toxicity, and oxidative stress injury. The bio-molecules absorbed on the surface may also help in the antimicrobial activities of CuO-NPs.

The CuO-NPs (200 g mL^−1^ concentration) exhibited superior antifungal activities. Qamar et al. (2020) showed reasonable results for the antifungal activity of CuO-NPs against *Trichophyton rubrum*. Rabiee et al. (2020) synthesized *Achillea millefolium* extract-mediated CuO-NPs and reported significant *in vitro* antifungal activities against four different fungi. The biosynthesized CuO-NPs showed effective antifungal activities owing to the entering of CuO-NPs on fungal membranes and negatively effect the cell divisions *via* strong interaction on the respiratory chains.

The use of CuO-NPs (100 mg L^−1^) resulted in the improvement of plant growth attributes as copper-based nano-fertilizer. The specific doses of CuO-NPs can play a remarkable role in plant growth promotion are advocated by several researchers (Singh et al., 2018; Rajput et al., 2022) due to the increase bio-availability of Cu^2+^ which led to accelerating the mobilization of food reserves during germination, greater activation of copper enzymes such as cytochrome C oxidase, *etc.* CuO-NPs in the optimum dose can significantly influence the plant growth and act as efficient nano-fertilizers.

## 5 Conclusion

In conclusion, we present a straightforward, quick, and environmentally friendly method for producing CuO-NPs with exceptional antibacterial properties. Different approaches have been applied to clarify the size, shape, composition, and stability, and the findings demonstrate that the synthesized nanoparticles are very stable and monoclinic, with the largest particles falling within the size range of 28 nm in diameter. The CuO-NPs may function as a potent bactericide and fungicide that may be employed to combat plant infections as a result of the positive results. With the right toxicological information, the greenly produced CuO-NPs have a large potential and may be used for a variety of tasks, including food processing and control, biomedical forms, product packaging, and more. According to the observations of this study, CuO-NPs are a novel class of antimicrobial agents that may be developed and applied in sustainable agriculture.

## Data Availability

The datasets presented in this study can be found in online repositories. The names of the repository/repositories and accession number(s) can be found below: https://www.ncbi.nlm.nih.gov/-, MK773873.

## References

[B1] Abboud Y. Saffaj T. Chagraoui A. Bouari A. El. Brouzi K. Tanane O. (2014). Biosynthesis, characterization and antimicrobial activity of copper oxide nanoparticles (CONPs) produced using Brown alga extract (*Bifurcariabifurcata*). Appl. Nanosci. 4, 571–576. 10.1007/s13204-013-0233-x

[B2] Abdulhameed A. S. Jawad A. H. Mohammad A. K. T. (2019). Synthesis of chitosan-ethylene glycol diglycidyl ether/TiO2 nanoparticles for adsorption of reactive orange 16 dye using a response surface methodology approach. Bioresour. Technol. 293, 122071. 10.1016/j.biortech.2019.122071 31491651

[B3] Ali M. Ijaz M. Ikram M. Ul-Hamid A. Avais M. Anjum A. A. (2021). Biogenic synthesis, characterization and antibacterial potential evaluation of copper oxide nanoparticles against *Escherichia coli* . Nanoscale Res. Lett. 16, 148. 10.1186/s11671-021-03605-z 34542713 PMC8452814

[B4] Amin F. Khattak B. Alotaibi A. Qasim M. Ahmad I. Ullah R. (2021). Green synthesis of copper oxide nanoparticles using *Aervajavanica* leaf extract and their characterization and investigation of *invitro* antimicrobial potential and cytotoxic activities. Evidence-Based Complemet. Altern. Med. 5589703, 1–12. 10.1155/2021/5589703 PMC8235967 34239581

[B5] Bhavyasree P. G. Xavier T. S. (2022). Green synthesised copper and copper oxide-based nanomaterials using plant extracts and their application in antimicrobial activity: Review. Curr. Res. Green Sustain. Chem. 5, 100249. 10.1016/j.crgsc.2021.100249

[B6] Buazar F. Sweidi S. Badri M. Kroushawi F. (2019). Biofabrication of highly pure copper oxide nanoparticles using wheat seed extract and their catalytic activity: A mechanistic approach. Green Process. Synthesis 8, 691–702. 10.1515/gps-2019-0040

[B7] Bukhari S. I. Hamed M. M. Al-Agamy M. H. Gazwi H. S. S. Radwan H. H. Youssif A. M. (2021). Biosynthesis of copper oxide nanoparticles using Streptomyces MHM38 and its biological applications. Hindawi J. Nanomater. 6693302, 1–16. 10.1155/2021/6693302

[B8] Chandra S. Kumar A. Kumar T. (2014). Synthesis and characterization of copper nanoparticles by reducing agent. J. Saudi Chem. Soc. 18 (2), 149–153. 10.1016/j.jscs.2011.06.009

[B9] Cheng G. Walker A. R. H. (2010). Transmission electron microscopy characterization of colloidal copper nanoparticles and their chemical reactivity. Anal. Bioanal. Chem. 396, 1057–1069. 10.1007/s00216-009-3203-0 19841909

[B10] Davaeifar S. Modarresi M. H. Mohammadi M. Hashemi E. Shafiei M. Maleki H. (2019). Synthesizing, characterizing, and toxicity evaluating of phycocyanin-ZnO nanorod composites: A back to nature approaches. Colloids Surfaces B Biointerfaces B 175, 221–230. 10.1016/j.colsurfb.2018.12.002 30537618

[B11] Garg K. K. Jain D. Rajpurohit D. Kushwaha H. S. Daima H. K. Stephen B. J. (2022). Agricultural significance of silica nanoparticles synthesized from a silica SolubilizingBacteria. Comments Inorg. Chem. 42 (4), 209–225. 10.1080/02603594.2021.1999234

[B12] Grigore M. E. Biscu E. R. Holban A. M. Gestal M. C. Grumezescu A. M. (2016). Methods of synthesis, properties and biomedical applications of CuO nanoparticles. Pharm. (Basel) 9 (4), 75. 10.3390/ph9040075 PMC5198050 27916867

[B13] Hidangmayum A. Debnath A. Guru A. Singh B. N. Upadhyay S. K. Dwivedi P. (2022). Mechanistic and recent updates in nano-bioremediation for developing green technology to alleviate agricultural contaminants. Int. J. Environ. Sci. Techno., 1–26. 10.1007/s13762-022-04560-7 PMC9521565 36196301

[B14] Ida K. Sugiyama Y. YukiChujyo Y. Tomonari M. Tomoharu Tokunaga T. Sasaki K. (2010). *In-situ* TEM studies of the sintering behavior of copper nanoparticles covered by biopolymer nanoskin. J. Electron Microsc. 59, 75–80. 10.1093/jmicro/dfq055 20573747

[B15] Jain N. Bhargava A. Tarafdar J. C. Singh S. K. Panwar J. (2012). A biomimetic approach towards synthesis of zinc oxide nanoparticles. Appl. Microbiol. Biotechnol. 97 (2), 859–869. 10.1007/s00253-012-3934-2 22382164

[B16] Jain D. Kour R. Bhojiya A. A. Meena R. H. Singh A. Mohanty S. R. (2020). Zinc tolerant plant growth promoting bacteria alleviates phytotoxic effects of zinc on maize through zinc immobilization. Sci. Rep. 10, 13865. 10.1038/s41598-020-70846-w 32807871 PMC7431563

[B17] Jain D. Kushwaha H. S. Rathore K. S. Stephen B. J. Daima H. K. Jain R. (2022). Fabrication of iron oxide nanoparticles from ammonia vapor and their importance in plant growth and dye degradation. Part. Sci. Technol. 40, 97–103. 10.1080/02726351.2021.1929601

[B18] Jain R. Bohra N. Singh R. K. Upadhyay S. K. Srivastava A. K. Rajput V. D. (2022). “Nanomaterials for plants: From ecophysiology to signaling mechanisms and nutrient uptake,” in The role of nanoparticles in plant nutrition under soil pollution. Sustainable plant nutrition in a changing world. Editors Rajput V. D. Verma K. K. Sharma N. Minkina T. (Cham: Springer). 10.1007/978-3-030-97389-6-8

[B19] Janda J. M. Abbott S. L. (2007). 16S rRNA gene sequencing for bacterial identification in the diagnostic laboratory: Pluses, perils, and pitfalls. J. Clin. Microbiol. 45 (9), 2761–2764. 10.1128/JCM.01228-07 17626177 PMC2045242

[B20] John M. S. Nagoth J. A. Zannotti M. Giovannetti R. Mancini A. Ramasamy K., P. (2021). Biogenic synthesis of copper nanoparticles using bacterial strains isolated from an antarctic consortium associated to a psychrophilic marine ciliate: Characterization and potential application as antimicrobial agents. Mar. Drugs 19 (5), 263. 10.3390/md19050263 34066868 PMC8151786

[B21] Keabadile O. P. Aremu A. O. Elugoke S. E. Fayemi O. E. (2020). Green and traditional synthesis of copper oxide nanoparticles-comparative study. Nanomater. (Basel) 10 (12), 2502. 10.3390/nano10122502 PMC7764311 33327366

[B22] Kimber R. L. Bagshaw H. Smith K. Buchanan D. M. Coker V. S. Cavet J. S. (2020). Biomineralization of Cu 2S nanoparticles by geobactersulfurreducens. Appl. Environ.Microbiol. 86, 009677–e1020. 10.1128/AEM.00967-20 PMC7480366 32680873

[B23] Krishna B. A. Kumar P. N. Prema P. (2020). Green synthesis of copper oxide nanoparticles using *Cinnamomum malabatrum* leaf extract and its antibacterial activity. Indian J. Chem. Technol. 27, 525–530.

[B24] Mahboub H. H. Rashidian G. Hoseinifar S. H. Kamel S. Zare M. Ghafarifarsani H. (2022). Protective effects of Allium hirtifolium extract against foodborne toxicity of Zinc oxide nanoparticles in Common carp (*Cyprinus carpio*). Comp. Biochem. Physiol. Part C Toxicol. Pharmacol. 257, 109345. 10.1016/j.cbpc.2022.109345 35429652

[B25] Makhluf S. Dror R. Nitzan Y. Abramovich R. J. Gedanken A. (2005). Microwave-assisted synthesis of nanocrystalline MgO and its use as a bacteriocide. Adv. Funct. Mater. 15, 1708–1715. 10.1002/adfm.200500029

[B26] Mohsen J. Zahra B. (2008). Protein nanoparticle: A unique system as drug delivery vehicles. Afr. J. Biotechnol. 7, 4926.

[B27] Nagaraj E. Karuppannan K. Shanmugam P. Venugopal S. (2019). Exploration of biosynthesized copper oxide nanoparticles using pterolobiumhexapetalum leaf extract by photocatalytic activity and biological evaluations. J. Clust. Sci. 30, 1157–1168. 10.1007/s10876-019-01579-8

[B28] Nardella M. I. Fortino M. Barbanente A. Natile G. Pietropaolo A. Arnesano F. (2022). Multinuclear metal-binding ability of the N-terminal region of human copper transporter Ctr1: Dependence upon pH and metal oxidation state. Front. Mol. Biosci. 9, 897621. 10.3389/fmolb.2022.897621 35601835 PMC9117721

[B29] Qamar H. Rehman S. Chauhan D. K. Tiwari A. K. Upmanyu V. (2020). Green synthesis, characterization and antimicrobial activity of copper oxide nanomaterial derived from momordica charantia. Int. J. Nanomed. 15, 2541–2553. 10.2147/IJN.S240232 PMC7170629 32368039

[B30] Rabiee N. Bagherzadeh M. Kiani M. Ghadiri A. M. Etessamifar F. Jaberizadeh A. H. (2020). Biosynthesis of copper oxide nanoparticles with potential biomedical applications. Int. J. Nanomed. 15, 3983–3999. 10.2147/IJN.S255398 PMC7294052 32606660

[B31] Rajput V. D. Minkina T. Fedorenko A. Chernikova N. Hassan T. Mandzhieva S. (2021a). Effects of zinc oxide nanoparticles on physiological and anatomical indices in spring barley tissues. Nanomaterials 11, 1722. 10.3390/nano11071722 34208886 PMC8307126

[B32] Rajput V. D. Singh A. Minkina T. Rawat S. Mandzhieva S. Sushkova S. (2021b). Nano-enabled products:challenges and opportunities for sustainable agriculture. Plants 10, 2727. 10.3390/plants10122727 34961197 PMC8707238

[B33] Rajput V. D. Minkina T. Upadhyay S. K. Kumari A. Ranjan A. Mandzhieva S. (2022). Nanotechnology in the restoration of polluted soil. Nanomaterials 12, 769. 10.3390/nano12050769 35269257 PMC8911862

[B34] Sagadevan S. Koteeswari P. (2015). Analysis of structure, surface morphology, optical and electrical properties of copper nanoparticles. J. Nanomed. Res. 2 (5), 133–136. 10.15406/jnmr.2015.02.00040

[B35] Sankar R. Manikandan P. Malarvizhi V. Fathima T. ShivashangariRavikumar K. S,V. (2014). Green synthesis of colloidal copper oxide nanoparticles using Carica papaya and its application in photocatalytic dye degradation. Spectrochim. Acta Part A Mol. Biomol. Spectrosc. 121, 746–750. 10.1016/j.saa.2013.12.020 24388701

[B36] Shantkriti S. Rani P. (2014). Biological synthesis of Copper nanoparticles using Pseudomonas. fluorescens. Int. J. Curr. Microbiol. Appl. Sci. 3 (9), 374–383.

[B37] Sharma M. Sharma A. Majumder S. (2020). Synthesis, microbial susceptibility and anti-cancerous properties of copper oxide nanoparticles-review. Nano Expres 1 (1), 012003. 10.1088/2632-959x/ab9241

[B38] Singh J. Dutta T. Kim K. H. Rawat M. Samddar P. Kumar P. (2018). 'Green' synthesis of metals and their oxide nanoparticles: Applications for environmental remediation. J. Nanobiotechnol. 16 (1), 84. 10.1186/s12951-018-0408-4 PMC6206834 30373622

[B39] Singh J. Kumar V. Kim K. H. Rawat M. (2019). Biogenic synthesis of copper oxide nanoparticles using plant extract and its prodigious potential for photocatalytic degradation of dyes. Environ. Res. 177, 108569. 10.1016/j.envres.2019.108569 31352301

[B40] Sobha K. Surendranath K. Meena V. K. Jwala T. Swetha N. Latha K. S. M. (2010). Emerging trends in nanobiotechnology. J. Biotechnol. Mol. Rev. 5, 01–12.

[B41] Sukhwal A. Jain D. Joshi A. Rawal P. Kushwaha H. S. (2017). Biosynthesized silver nanoparticles using aqueous leaf extract of *Tagetuspatula* L. and evaluation of their antifungal activity against phytopathogenic fungi. IET Nanobiotechnol. 11, 531–537. 10.1049/iet-nbt.2016.0175 28745285 PMC8676471

[B42] Sukumar S. Rudrasenan A. PadmanabhanNambiar D. (2020). Green-Synthesized rice-shaped copper oxide nanoparticles using *Caesalpinia bonducella* seed extract and their applications. ACS-Omega 5 (2), 1040–1051. 10.1021/acsomega.9b02857 31984260 PMC6977032

[B43] Tavakoli S. Kharaziha M. Ahmadi S. (2019). Green synthesis and morphology dependent antibacterial activity of copper oxide nanoparticles. J. Nanostruct. 9 (1), 163–171. 10.22052/JNS.2019.01.018

[B44] Tiwari M. Jain P. Hariharapura R. Kashinathan N. Bhat B. Nayanabhirama U. (2016). Biosynthesis of copper nanoparticles using copper-resistant Bacillus cereus, a soil isolate. Process Biochem. 51, 1348–1356. 10.1016/j.procbio.2016.08.008

[B45] Tshireletso P. Ateba C. N. Fayemi O. E. (2021). Spectroscopic and antibacterial properties of CuONPs from orange, lemon and tangerine peel extracts: Potential for combating bacterial resistance. Molecules 26, 586. 10.3390/molecules26030586 33499352 PMC7865892

[B46] Upadhyay S. K. Devi P. Kumar V. Pathak H. K. Kumar P. Rajput V. D. (2023). Efficient removal of total arsenic (As^3+/5+^) from contaminated water by novel strategies mediated iron and plant extract activated waste flowers of marigold. Chemosphere 313, 137551. 10.1016/j.chemosphere.2022.137551 36521746

[B47] Zhao H. Maruthupandy M. Al-mekhlafi F. A. Chackaravarthi G. Ramachandran G. Chelliah C. K. (2022). Biological synthesis of copper oxide nanoparticles using marine endophytic actinomycetes and evaluation of biofilm producing bacteria and A549 lung cancer cells. J. King Saud Univ. - Sci. 34 (3), 101866. 10.1016/j.jksus.2022.101866

