# Supplementary material for: Bacteria assisted green synthesis of copper oxide nanoparticles and their potential applications as antimicrobial agents and plant growth stimulants
Source: Front Chem. 2023 Apr 7;11:1154128. doi: 10.3389/fchem.2023.1154128 (PMC10119401; doi:10.3389/fchem.2023.1154128)
Supplement: Supplementary file 1 [file DataSheet1.docx]

Supplementary Material

Optimization of bacterial-assisted synthesis of copper oxide nanoparticles and their potential applications as antibacterial agent and plant growth stimulants

Deepak Singh^1^, Devendra Jain^1*^, Deepak Rajpurohit^1^, Gajanand Jat^2^, Himmat Singh Kushwaha^3^, Abhijeet Singh^4^, Santosh R. Mohanty^5^, Mohammad Khalid Al-Sadoon^6^, Wajid Zaman^7^, Sudhir K. Upadhyay^8^

^1^Department of Molecular Biology and Biotechnology, Maharana Pratap University of Agriculture and Technology, Udaipur, India

^2^Department of Soil Science and Agricultural Chemistry, Maharana Pratap University of Agriculture and Technology, Udaipur, India

^3^Material Research Centre, Malviya National Institute of Technology, Jaipur, India

^4^Department of Biosciences, Manipal University Jaipur, Jaipur, India

^5^All India Network Project on Soil Biodiversity-Biofertilizers, ICAR-Indian Institute of Soil Science, Bhopal- 462038, India

^6^Department of Zoology, College of Science, King Saud University, PO Box 2455, Riyadh,11451, Saudi Arabia.

^7^Department of Life Sciences, Yeungnam University, Gyeongsan, South Korea

^8^Department of Environmental Science, V. B. S. Purvanchal University, Jaunpur-222003, India

# * Correspondence: Dr. Devendra Jain [devroshan@gmail.com](mailto:devroshan@gmail.com); [devendrajain@mpuat.ac.in](mailto:devendrajain@mpuat.ac.in)

Supplementary Material should be uploaded separately on submission. Please include any supplementary data, figures and/or tables.

# Supplementary Tables

For more information on Supplementary Material and for details on the different file types accepted, please see [here](https://www.frontiersin.org/guidelines/author-guidelines#supplementary-material).

## Supplementary table S1: Determination of Minimum Inhibitory Concentration (MIC) of bacterial isolates (+ indicates the growth on the medium)

| Cu^2+^ concentration | ZTB15 | ZTB24 | ZTB28 | ZTB29 |
| --- | --- | --- | --- | --- |
| **2.5 Mm** | + | + | + | + |
| **5 Mm** | + | + | + | + |
| **7.5 mM** | + | + | + | + |
| **10 mM** | + | + | + | + |
| **12.5 mM** | + | + | + | + |
| **15 mM** | + | + | + | + |
| **17.5 mM** | + | + | + | + |
| **22.5 mM** | - | - | - | + |
| **25.0 mM** | - | - | - | - |

**1.2 Supplementary table S2: Biochemical and Plant Growth Promoting Activities of copper tolerant ZTB29**

| **PGPR Activity** | **ZTB29** |
| --- | --- |
| Starch Hydrolysis | + |
| Citrate Utilization | + |
| Nitrate Reduction | - |
| Gelatin liquefaction | - |
| Catalase Activity | + |
| Oxidase Activity | - |
| IAA Production (µg/mL) | 12.54 |
| ACC Deaminase Activity | + |
| Ammonia Production (µg/mL) | 1.45±0.86 |
| HCN Production | - |
| GA3 (µg/mL) | 28.10±1.01 |
| Phosphate Solublization Index | 3.85± 0.04 |
| Potassium Solublization Index | 8.00± 0.10 |
| Silica Solublization Index | 2.30± 0.01 |
| Phytase Production Index | 11.42±0.01 |
| Siderophore Index (Z/C) | 2.00±0.60 |
| EPS production | + |
| Gluconic caid production | + |
| PCR based amplification of *czcD* gene | amplified |

**+ Positive; ++ Medium Positive; +++ High Positive; - Negative; Data is presented as means of 3 replicates ± S.D (standard deviation)**

**1.3 Table Supplementary table S3: FTIR peak analysis of bacterial assisted CuO-NPs**

| **Peak Position** | **Functional group** | **Compound class** |
| --- | --- | --- |
| 3415 cm^−1^ | O-H stretching | Alcohol |
| 2958 cm^−1^ | O-H stretching | Carboxylic acid |
| 2925 cm^−1^ | C=C stretching | Alkane |
| 2854 cm^−1^ | N-H stretching, | Amine salt |
| 2364-2337 cm^−1^ | O=C=O stretching | CO_2_ |
| 1657 cm^−1^ | C=C stretching | Alkane |
| 1542 cm^−1^ | N-O stretching | Nitro compound |
| 1446 cm^−1^ | C-H bending, C-N stretching | Alkane |
| 1384 cm^−1^ | O–H bending | Carboxylic acid |
| 1233 cm^−1^ | C-N stretching | Amine |
| 1145 cm^−1^ | C-O stretching | Ester |
| 1076 cm^−1^ | C-F stretching | Fluoro compound |
| 1054 cm^−1^ | Cu–O stretching | Monoclinic |
| 923 cm^−1^ | C=C bending | Disubstituted |
| 670 cm^−1^ | C-H bending | Monosubstituted |
| 608 cm^−1^ | Cu–O stretching | Monoclinic |

# Supplementary Figures

2.1. Supplementary Figure S1: UV-Vis spectra of Copper sulphate solution


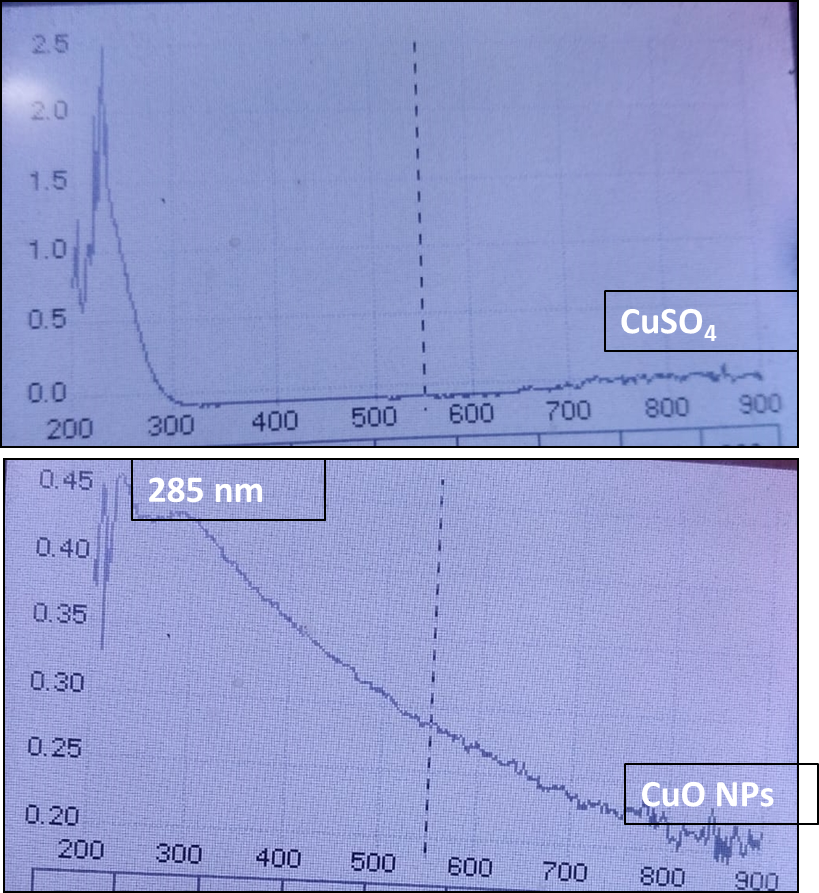


2.2. Supplementary Figure S1: FTIR spectra of bacterial extract used for synthesis of CuO-NPs


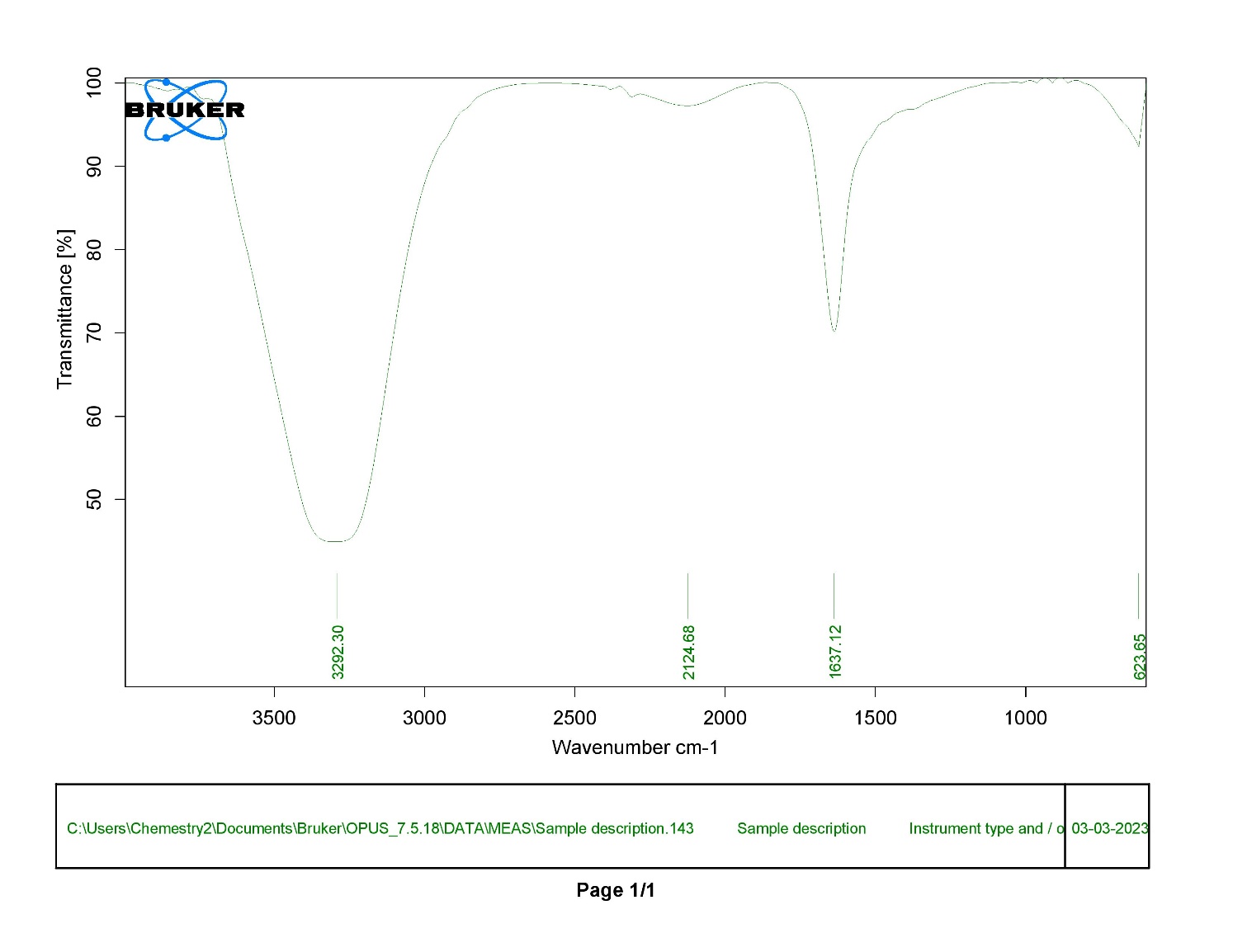


**
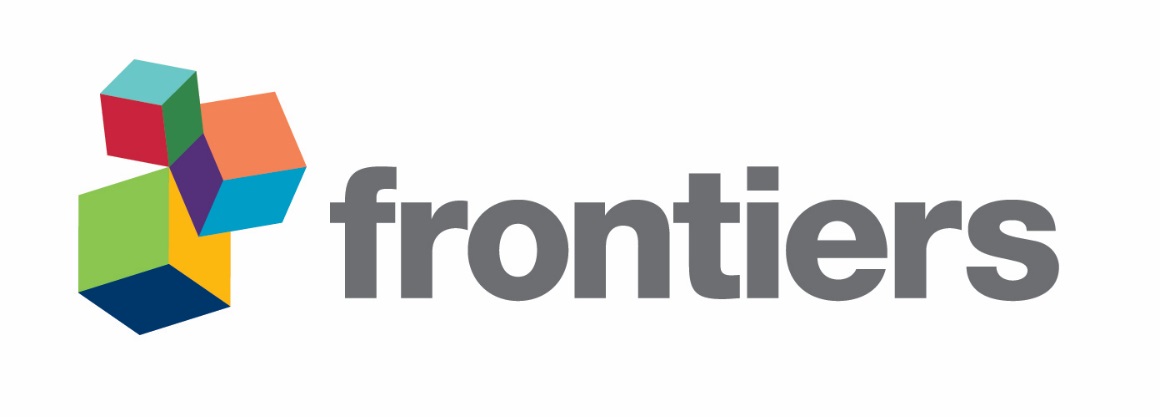
**
